# ChatGPT indicates the path and initiates the research to open up the black box of artificial intelligence

**DOI:** 10.1097/JS9.0000000000000701

**Published:** 2023-10-11

**Authors:** Chiranjib Chakraborty, Manojit Bhattacharya, Md. Aminul Islam, Govindasamy Agoramoorthy

**Affiliations:** a Department of Biotechnology, School of Life Science and Biotechnology, Adamas University, Kolkata, West Bengal; b Department of Zoology, Fakir Mohan University, Vyasa Vihar, Balasore, Odisha; c Honeybee Population Health Foundation, Chennai, India; d Advanced Molecular Lab, Department of Microbiology, President Abdul Hamid Medical College, Karimganj, Kishoreganj; e COVID-19 Diagnostic Lab, Department of Microbiology, Noakhali Science and Technology University, Noakhali, Bangladesh; f College of Pharmacy and Health Care, Tajen University, Yanpu, Pingtung, Taiwan

*Dear Editor*,

A recent article by Chiu *et al*.^1^ informed the role of artificial intelligence (AI)-enabled colonoscopy for the early detection of colorectal cancer was published in this journal. At the same time, another article recently informed about the AI-assisted ChatGPT’s (Chat Generative Pre-trained Transformer) application in medical science was also published in this journal^2^. Both of these two articles about the application of AI are very timely.

Researchers have lately used approaches of AI during the pandemic to identify and characterize coronavirus disease 2019 (COVID-19) cases using computed tomography (CT) imaging data^3^. AI not only supports detecting COVID-19 cases, but also has supported several physicians in diagnosing diseases from oncology to ophthalmology. The first AI-based software was approved for marketing to Arterys Inc. (San Francisco, USA) by the Food and Drug Administration (FDA) on November 2016. The software was developed to analyze cardiovascular images using a deep learning algorithm^4^. Subsequently, the U.S. FDA approved several AI products for medical diagnosis^4^. Recent AI-enabled ChatGPT, an LLM (Large Language Model) developed by OpenAI (San Francisco, California, U.S.), has gained the attention of the academic community, researchers, and physicians and is quickly popularized. Over one million users have been reported to use the ChatGPT within 5 days after its release^5^. Therefore, AI has entered every segment of human endeavor, such as health, engineering, and science. However, the proper and complete mechanism of AI is still unclear and is called the black box of AI. Therefore, it is an urgent need to understand the mechanism of AI accurately.

In this direction, the first question raised by Alan Turing was ‘Can machines think?’^6^. Turing proposed an ‘imitation game,’ which was a text-based interaction between humans and a computer. The game was popularized as the Turing test. However, the test was considered too vague because it concentrated on deception rather than explaining the proper intelligent behavior^7^. However, due to the advancement of AI, researchers are trying to illustrate different mechanisms of AI through various algorithms, such as artificial neural networks (ANN), convolutional ANNs, and reinforcement learning that were inspired mainly by neuroscience as it guides to explicate the algorithms. One significant example is the neural networks inspired and designed by the mechanisms in brain^8,9^. Unfortunately, such networks generated by the neurons are opaque as the brain itself. During testing, it was observed that the information was diffused, so it wasn’t straightforward to decipher it^10^. However, the opacity model of AI, called the black box model, shows concern about understanding the AI because we still don’t know how the black box model is governed. Till today, computer scientists are unable to illustrate the black box model. Computing costs and describing the best results is extremely complicated without ambiguity. Therefore, it is one of the significant challenges in current computer science for over a decade to describe the black box^11^. Therefore, scientists are asking a significant question: Can the black box of AI be opened? On the same line, Castelvecchi raised a similar query in Nature News^10^.

After the recent release of the AI-enabled ChatGPT, it was popularized and applied in various fields of medical science, research, essay writing, etc. All at once, the LLM has created a significant debate about the models’ strengths and weaknesses. In this context, however, it is necessary to understand the mechanism that causes the behavior of the AI-enabled LLM. It is also essential to predict the underlying mechanism of the LLM since researchers are already thinking in this direction^12^. They are trying to solve the decade’s most promising challenge: unraveling the black box of AI.

To better understand the algorithms used by ANN guided by neuroscience, the NIH BRAIN initiative and other scientists have proposed the NeuroAI model to comprehend neural computation recently^9^. It may reveal fundamental intelligence and the mechanism of the next revolution in AI. However, in the near future, the mechanism of the black box might decipher with the proper understanding of the NeuroAI model. The present technological revolution of LLMs has again initiated the beginning of thanking how essential and urgent it is to reveal the black box. However, the ChatGPT has initiated the research to open up the black box of AI with the NeuroAI model, which may enhance the understanding of NeuroAI connection. In this direction, all governments and policymakers worldwide should understand the fact that AI research is essential, which is humanity’s technological future, which should follow some necessary steps. First, training the next-generation AI researchers with equal computer science and neuroscience knowledge is necessary. At the same time, it is also crucial to expand research on neural computation guided by fundamental theoretical and experimental basis. This may eventually leads to the unraveling of the mechanisms of AI to benefit humanity.

## Ethical approval

Not applicable.

## Sources of funding

Not applicable.

## Author contribution

C.C.: conceptualization, data curation, investigation, writing – original draft, –review, and editing; M.B.: validation; Md.A.I.: validation; G.A.: validation, review, and editing. All authors critically reviewed and approved the final version of the manuscript.

## Conflicts of interest disclosure

All authors report no conflicts of interest relevant to this article.

## Research registration unique identifying number (UIN)

1. Name of the registry: Not applicable.
2. Unique identifying number or registration ID: Not applicable.
3. Hyperlink to your specific registration (must be publicly accessible and will be checked): Not applicable.

## Guarantor

Md. Aminul Islam, COVID-19 Diagnostic Lab, Department of Microbiology, Noakhali Science and Technology University, Noakhali 3814, Bangladesh; E-mail: aminulmbg@gmail.com

## Data availability statement

The data in this correspondence article is not sensitive in nature and is accessible in the public domain. The data is therefore available and not of a confidential nature.

## Provenance and peer review

Not commissioned, internally peer-reviewed.
